# CpG Island Methylator Phenotype—A Hope for the Future or a Road to Nowhere?

**DOI:** 10.3390/ijms23020830

**Published:** 2022-01-13

**Authors:** Karpiński Paweł, Sąsiadek Maria Małgorzata

**Affiliations:** Depatment of Genetics, Wroclaw Medical University, 50-368 Wrocław, Poland; maria.sasiadek@umw.edu.pl

**Keywords:** epigenetics, methylator phenotype, CIMP, DNA methylation

## Abstract

The CpG island methylator phenotype (CIMP) can be regarded as the most notable emanation of epigenetic instability in cancer. Since its discovery in the late 1990s, CIMP has been extensively studied, mainly in colorectal cancers (CRC) and gliomas. Consequently, knowledge on molecular and pathological characteristics of CIMP in CRC and other tumour types has rapidly expanded. Concordant and widespread hypermethylation of multiple CpG islands observed in CIMP in multiple cancers raised hopes for future epigenetically based diagnostics and treatments of solid tumours. However, studies on CIMP in solid tumours were hampered by a lack of generalisability and reproducibility of epigenetic markers. Moreover, CIMP was not a satisfactory marker in predicting clinical outcomes. The idea of targeting epigenetic abnormalities such as CIMP for cancer therapy has not been implemented for solid tumours, either. Twenty-one years after its discovery, we aim to cover both the fundamental and new aspects of CIMP and its future application as a diagnostic marker and target in anticancer therapies.

## 1. Introduction

**Figure 1:**
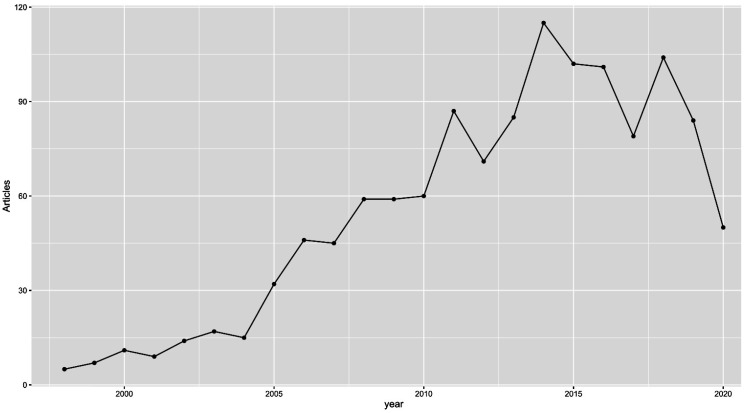
Graph depicting the number of PubMed articles published on CIMP each year between 1999 and 2020. We used the RISmed R package for analysis and “methylator phenotype OR CIMP”as the search term.

Over the past 30 years, DNA methylation has been extensively studied and defined as a key repressive epigenetic mark [1]. More and more, studies have indicated that processes of hypermethylation and hypomethylation are deeply involved in carcinogenesis. Additionally, a significant number of cancer-specific aberrant methylation events have been identified, and their usefulness in cancer diagnostics is becoming increasingly clear, as many of them appear long before the malignant transition [2]. Moreover, the reversibility of epigenetic changes, including DNA methylation, attracted extensive attention, which resulted in the introduction of nine drugs tagreting epigenetic enzymes that have been approved by the FDA (azacitidine, decitabine, tazemetostat, ivosidenib, enasidenib, vorinostat, panobinostat, belinostat and romidepsin) [3]. The discovery of a subgroup of tumours with frequent and concurrent hypermethylation of CpG islands (CIMP—the CpG island methylator phenotype) in the late 1990s perfectly matched the trend of interest in DNA methylation; we believe that CIMP may have been one of the most popular and disputed topics in cancer epigenetics at that time [4]. Considering the significant number of hypermethylation events, CIMP-positive tumours seemed to have perfect DNA methylation constitution to be diagnosed early and treated efficiently by demethylating drugs [5]. Unfortunately, none of that has ever materialised. As visualised in Figure 1, the number of papers published annually on CIMP has dropped significantly since 2017. In this paper, we attempt to show the historical context of CIMP and describe the current status of research on CIMP in various cancers.

## 2. DNA Methylation

DNA methylation is an epigenetic mark that consists of the covalent transfer of a methyl group to the C-5 position of the cytosine ring of DNA by DNA methyltransferases (DNMTs) [6]. The process is heritable, but also reversible. Studies on mammals show that more than 98% of DNA methylation occurs in a CpG dinucleotide context in somatic cells, and up to a quarter is found in a non-CpG context in embryonic stem cells (ESCs), although it is possible at cytosines in any context of the genome. In mammals, most CG dinucleotides are methylated on cytosine residues, but CG dinucleotides within promoters are most commonly free from methylation [2]. Typically, DNA methylation disappears during zygote formation and is then restored in the embryo around the moment of implantation. As DNA methylation is a key factor of genomic imprinting, X-chromosome inactivation and a range of other processes, it plays a vital role in normal development; however, its significance for the development and control of neoplasms has been confirmed, as well [7]. There is evidence that DNA methylation provides the critical signal that is necessary for cancer initiation, proliferation and survival in the early phase [8]. Global DNA hypomethylation and locus-specific hypermethylation of CpG islands (CGIs) are considered the epigenetic hallmarks of cancer. With experimental research models, it has become possible to explore the impact of the DNA methylation mechanism on gene transcription. Hypomethylation is mostly caused by the loss of methylation at the repeat elements that are normally heavily methylated, such as satellites (e.g., SAT2) and retrotransposons (e.g., ALUs, LINEs). This loss triggers genomic instability and activation of oncogenes [9]. Locus-specific hypermethylation is typically found in promoter CGIs of diverse genes; it leads to transcriptional silencing which can be inherited. In another mechanism, hypermethylation itself may physically block the process of binding of transcriptional regulators and genes [9]. Significant effects are also associated with the methylated DNA’s role in chromatin condensation due to interactions with various other epigenetic modifications, such as the histone code, polycomb complexes, nucleosome positioning, noncoding RNA, and ATP-dependent chromatin remodelling proteins [10].

## 3. Discovery of CpG Island Methylator Phenotype (CIMP)

The two last decades of the 20th century witnessed immense progress in molecular biology, which contributed to the development in the 1990s of a new concept of analysing the epigenetic aspect of cancer aetiology [11]. The new hypothesis provided for exploration of two distinct genetic processes associated with neoplastic pathology: not only the aberrant changes in DNA sequence (mutations), but also changes in the DNA methylation pattern. Importantly, these mutations and methylation mechanisms were found to be mutually exclusive, indicating that they both brought equivalent selective advantages to affected cells [12].

Research on epigenetics in cancer has been significantly accelerated by the introduction in the early 1990s of numerous straightforward solutions dedicated to the detection of DNA methylation. In 1992, Frommer et al. introduced a ground-breaking sequencing approach to detect 5-methylcytosine residues based on the bisulfite-induced conversion of genomic DNA [13]. This paved the way for PCR and bisulphite-based methods to study the methylation status of the previously known locus or few loci: qualitative methylation-specific (MS)-PCR by Herman et al. (in 1996) and quantitative combined bisulfite restriction analysis (COBRA) by Xiong et al. in 1997 [14,15]. Although these techniques were very convenient in detecting methylation differences, they were limited to known genes because they required sequence information for the design of PCR primers [16]. In parallel, restriction enzyme-based techniques supporting the identification of de novo DNA methylation changes were introduced into the cancer field, including restriction landmark genomic scanning (RLGS) and methylation-sensitive arbitrarily primed PCR, both in 1997 [17,18]. More specifically, research on CIMP has been triggered by the restriction enzyme-based method developed in 1999 in Jean Pierre Issa lab, i.e., methylated CpG island amplification (MCA) [19]. MCA employed by Toyota et al. made it possible to identify 33 hypermethylated CpG islands (designated MINT–methylated in tumour) in colon cancer cell line (CaCo-2) [19]. In the same year, Toyota et al. employed MCA and MINTs to study DNA methylation changes in several dozens of colorectal cancers (CRC) and adenomas [20]. This led to the discovery of age-related and cancer-specific methylation patterns in CRC. More importantly, cancer-specific patterns of DNA methylation revealed the existence of a cluster of tumours with frequent and concurrent hypermethylation of MINTs. Toyota et al. has termed this phenomenon the CpG island methylator phenotype (CIMP). CIMP has been proposed by Toyota et al. to be a novel, alternative pathway of colorectal carcinogenesis [21]. CIMP-positive colon tumours displayed strong associations with proximal location, frequent tumour suppressor hypermethylation, hMLH1 gene hypermethylation and microsatellite instability. In the same year (1999), Toyota et al. reported on the identification of CIMP in gastric cancer (GC) [21]. GC CIMP-positive tumours shared some features with CRC CIMP, including frequent hMLH1 gene hypermethylation and microsatellite instability (MSI). Afterwards, in 2000, additional molecular correlates for CIMP-positive tumours were provided by Toyota et al. namely KRAS mutation enrichment and low TP53 mutation frequency when compared to CIMP-negative tumours [22].

In summary, the discovery of CIMP was possible due to the introduction of methods for rapid identification of de novo DNA methylation changes in cancer and powerful bisulphite-dependent PCR-based methods to study methylation of a relatively large number of separate loci. At that time, it was expected that CIMP may have profound molecular and pathological consequences in many cancers through concordant transcriptional inactivation of many important genes including tumour suppressors, anti-apoptotic genes and many others [23,24]. It was also believed that, due to the reversibility of DNA methylation, CIMP-positive tumours and neoplasms may become a target of effective chemotherapeutical intervention with the use of DNA methylation inhibitors [25].

## 4. Pre-Microarray Era—Lack of CIMP-Specific Markers

By the early 21st century, the question of the presence of CIMP in other cancers had been intensively explored [26,27,28,29]. Most studies published during this period utilised nonspecific, variable sets of target genes to draw a picture of CIMP in their cancer datasets. Frequently, methylation of MINTs (MINT1, MINT2 and MINT31) proposed by Toyota et al., in addition to tumour suppressor genes, (for example hMLH1, MGMT, TIMP-3, HIC1, HOXA11, APC, CDKN2A, RASSF1A) were examined [30]. Consequently, the existence of CIMP-positive tumours has been announced in many neoplasms, including ovarian, kidney, pancreatic, oesophageal, bladder, endometrial and liver cancers [28,30,31,32,33]. Meanwhile, few research groups have questioned the existence of CIMP in CRC due to the arbitrary guidelines used to define it. In 2003, Yamashita et al. observed gradual distribution of methylation of tumour-suppressors that followed Gaussian distribution without a clear cut-off between the CIMP-positive and CIMP-negative tumours [34]. They suggested that methylation accumulation observed in CRC tumours is an age-dependent gradual process rather than non-random cancer-specific aggregation. Importantly, they provided evidence that molecular differences between CIMP-positive and negative tumours vanished after the exclusion of MSI tumours from the analysis. This notion has been independently confirmed via study by Anacleto et al. in 2005. This suggested that the phenotype of MSI is a significant confounder that dominates the CIMP phenotype in microsatellite unstable CIMP-positive tumours [35].

At that time, MS-PCR was a widely used tool to study the methylation status of the above markers. MS-PCR was a rapid and sensitive approach; however, it suffered from false positives, low-throughput, PCR bias, and most importantly, it offered only qualitative output (methylated or hemimethylated or unmethylated status of CGI) [36]. The Eads et al. paper published in 2000 introduced a high-throughput quantitative methylation assay that utilised fluorescence-based real-time PCR (so-called MethyLight assay) [37]. This was a significant improvement when compared to MS-PCR that paved the way for the possibility to observe the methylation landscape of neoplasia in large sample sizes and greater detail, and, importantly, in a quantitative manner. Consequently, six years later, Weisenberger et al. used a MethyLight assay to quantitatively examine the methylation of 195 CGIs in nearly 300 CRC cases [38]. The results shown by Weisenberger et al. supported the existence of bimodal, non-scholastic distribution of CGI methylation in CRC, as well as provided a novel feature displayed by CIMP-positive subgroup–frequent BRAFV600E mutation. This work also provided a novel marker panel specifically designed to detect CIMP tumours (CACNA1G, IGF2, NEUROG1, RUNX3 and SOCS1) [38]. Since then, the so-called Weisenberger panel has dominated CIMP research in CRC. This emphasized the importance of using proper marker panel(s) to identify CIMP and the urgent need to find optimal markers to study CIMP outside CRC. Having the presumably right marker panel as a tool to identify CIMP in CRC, research groups started to explore large cohorts of CRC patients to try to find novel clinical and molecular correlates of CIMP and/or find the prognostic value of CIMP. Consequently, CIMP in CRC has been associated with older age, mucinous histology, and inversely associated with LINE-1 hypomethylation and chromosome 18q loss [39,40,41,42]. Although, in 2009, Kim et al. had indicated a poor prognosis of CIMP-positive/MSI−CRCs, it was found to be attributed to the presence of BRAF V600E mutation [43]. In the following studies, including a large meta-analysis published in 2014 by Juo et al., CIMP was independently associated with significantly worse prognosis in CRC patients [44]. In parallel, others (including our research group) attempted at finding a factor or factors that contribute to the introduction of CIMP in CRC tumours. A major focus has been placed on the presence of DNA viruses (JC-virus, papillomaviruses, adenoviruses and herpesviruses) or sequence variants in methyl-group metabolism [45,46,47]. Although initial research provided by Goel et al. in 2006 has provided evidence for the contribution of JC-virus expression to CIMP, this has not been confirmed for JC-virus or any other viral species in the subsequent studies [48,49]. To our best knowledge, no current study has attributed the expression of any viral or bacterial species to CIMP in CRC. However, a strong association between the presence of the Epstein–Barr virus and CIMP has been reported in gastric cancer in 2006 [50]. Subsequent studies have confirmed that EBV infection triggers extensive methylation to silence multiple tumour suppressor genes and is responsible for the occurrence of an EBV-specific CIMP phenotype in gastric cancer [51].

Similarly to studies on viral presence, many separate studies have found associations for single or multiple single nucleotide variants (SNVs) with CIMP. Predominantly, SNVs in methyl-group metabolism were examined in this context, including such genes as MTHFR (Methylene tetrahydrofolate Reductase), MTHFD1 (Methylene tetrahydrofolate Dehydrogenase 1), TYMS (Thymidylate Synthetase) and DNMT3B (DNA Methyltransferase 3 Beta) [52]. Two large independent studies reported rs1801131 in the MTHFR gene to be significantly associated with the occurrence of CIMP [47,53]. However, this has not been confirmed in the 3rd study—a relatively large project conducted by Dutch scientists [53]. There were numerous early reports on the implication of the MLH1 (−93G > A; rs1800734) variant with CIMP-positive CRCs; however, recent studies showed that rs1800734 may contribute to increased methylation of the MLH1 gene promoter and the induction of MSI in some CRC tumours. Still, the presence of rs1800734 does not explain the induction of CIMP [54,55]. In conclusion, there is still a lack of strong evidence that low-penetrance SNVs may drive colorectal tumours down the CIMP-positive pathway.

In parallel, CRCs with intermediate levels of DNA methylation and KRAS mutation enrichment were also identified (described as CIMP-low, CIMP2 or CIMP-intermediate) [41,56,57,58]. That added significant complexity to the already molecularly heterogeneous disease and was reflected by two independent CRC classifications proposed by Jeremy Jass in 2007 and Suji Ogino and Ajay Goel in 2008, respectively [59,60]. These classifications proposed the existence of five to six distinct molecular subgroups in CRC, depending on CIMP and MSI status.

While early 2000s research on CIMP in CRC—and to a lesser extent in GC—was dominating the field of cancer epigenetics, some groups also reported CIMP in other cancers, including pancreatic cancer, neuroblastoma, T-cell acute lymphoblastic leukemia, ovarian cancer, hepatocellular carcinoma, and neuroendocrine tumours of the gastrointestinal tract [61,62,63,64]. In contrast, there were reports on the lack of detection of CIMP in several cancers, including uveal melanoma, retinoblastoma and breast cancer [65,66,67]. It must be emphasized that all mentioned studies suffered from the arbitrary selection of methylation markers that frequently included some tumour suppressor genes and CRC-specific MINT markers. This was due to the lack of precise knowledge at that time about the methylation profile of genes in healthy human tissues. Consequently, there was uncertainty as to whether CIMP is a global phenomenon that can be detected using one set of markers, such as in the case of microsatellite instability [68].

## 5. Microarray and TCGA Era

Studies on CIMP in various cancers took off significantly with the adaptation of microarray hybridisation techniques from the gene expression and genomic fields to the profiling of genome-wide DNA methylation patterns [16]. One of the frequently used approaches in cancer studies at that time was methyl-DNA immunoprecipitation (MeDIP) which is based on pulldown of methylated DNA with antibodies specific for 5′-methylcytosine (5MeC) [69]. MeDIP coupled with microarray hybridisation enabled the detection of differentially methylated regions (genes) along the genome [70]. Nonetheless, it was the adaptation of the single nucleotide polymorphism (SNP) Illumina bead array platform that significantly accelerated studies on DNA methylation in cancer. Its first generation (Golden Gate) was announced in 2006 and made it possible to analyse the methylation state of 1536 specific CpG sites in 371 genes [71]. Subsequently, the 2nd generation of Illumina arrays from 2009 enabled the analysis of 27,000 CpG sites, located mainly in gene promoters [72]. However, the 3rd generation of Illumina methylation microarray (Infinium HumanMethylation450K), released in 2011, became the most popular method of methylation profiling in cancer studies [73]. Featuring 485,577 probes, the 450K array design was mainly focused on CGIs, regions adjacent to CGIs shores and shelves and sites important in protein-coding gene expression regulation surrounding the transcription start sites (−200 bp to −1500 bp, 5′-UTRs and first exon). Some probes on the 450K array were also located in the intergenic (open sea) regions and in repetitive elements (LINE-1 and Alu). In summary, the 450K array constituted a low-cost and relatively high-resolution tool for scientists to study DNA methylation-related phenomena, including hypermethylation and hypomethylation events in cancer. In the context of CIMP, the Illumina 450K array enabled unbiased low-resolution screening of the methylation of CGIs located inside or outside gene promoters. It is worth mentioning that the current 4th generation of Illumina methylation arrays (MethylationEPIC BeadChip) released in 2016 makes it possible to analyse over 850,000 CpG sites [74].

**Figure 2:**
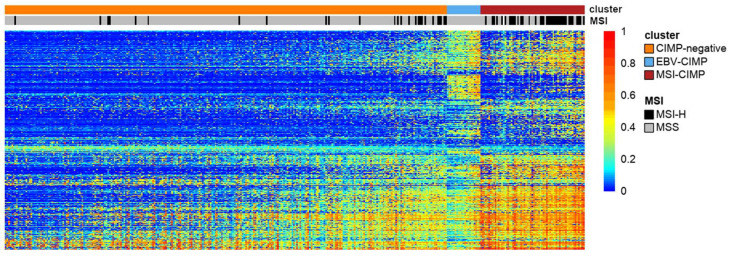
A heatmap representing the result of clustering of cancer-specific CGI methylation in gastric cancer based on TCGA Illumina 450K array data. Note the existence of two different clusters with high levels of GI methylation: MSI-associated (MSI-CIMP) and EBV-associated (EBV-CIMP). The heatmap was generated based on unsupervised clustering (COMMUNAL R package) of CpG probes located in the proximity (±500 bp) of transcription start sites (TSS) that displayed high cancer-specific methylation [79]. The extraction of CGI methylation, together with CIMP assignments, were described previously in our work [80].

The presence on the market of such a convenient tool as the Illumina 450K array was one of the conditions necessary to significantly accelerate studies on CIMP in various cancers. The second condition was met in 2005 when the Cancer Genome Atlas (TCGA) was launched [75]. TCGA created and published data for large cohorts of over 33 human tumour types along with their genomic profiles, including mRNA and miRNA expression, somatic single-nucleotide variation, copy number variations and DNA methylation (mainly using the Illumina 450K array). The presence of such a wide portfolio of genomic profiles enabled scientists to study CIMP in a wider context outside of epigenetics, for example, in the context of integrative molecular subtypes [76,77]. Figure 2 displays our visualisation of CGI methylation clusters in gastric cancer based on TCGA Illumina 450K array data. This is a unique tumour type, as it contains two separate CIMP clusters (MSI-associated and EBV-associated) [51].

TCGA studies have confirmed or found de novo CIMP in numerous cancers. Table 1 summarises discoveries on CIMP by TCGA in 28 tumour types. In brief, TCGA confirmed the presence of CIMP in colorectal and stomach cancers and provided evidence for CIMP in dozens of other cancers [78]. The majority of analysed cancer types display smaller or larger subgroups of tumours with significantly elevated methylation levels compared to the remaining samples.

## 6. Post-TCGA Era

**Figure 3:**
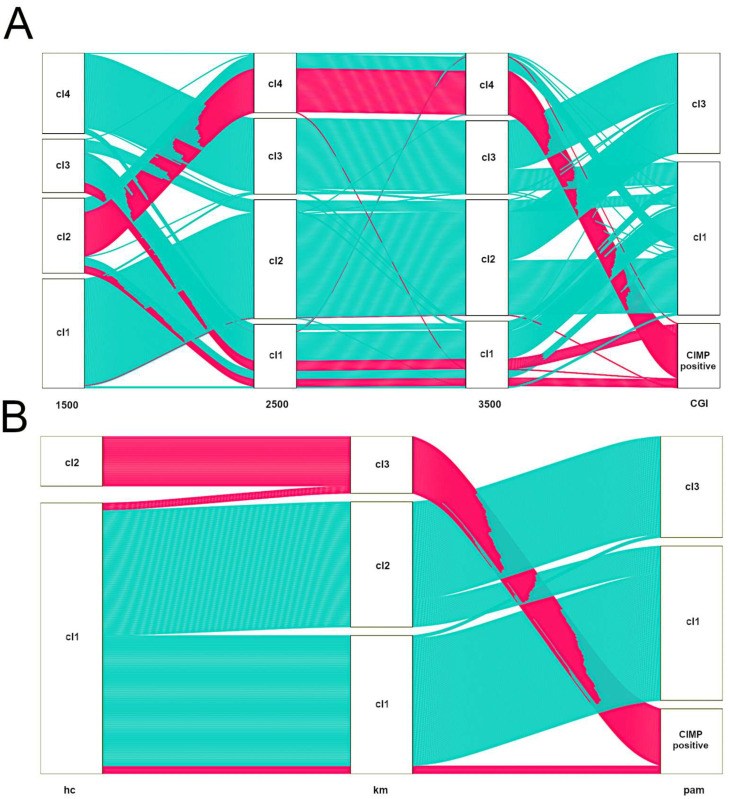
Alluvial diagrams illustrating the influence of choice of variable selection strategy (**A**) or clustering algorithm (**B**) on CIMP-positive assignment in colon cancer (TCGA dataset). In all cases, we used the consensus clustering approach with Euclidean distance and average linkage [120]. Consensus clustering was run using 80% sample resampling, a maximum evaluated k of 8 and 1000 resamplings. (**A**). Samples were clustered by pam (partitioning around medoids). All together, 1500 or 2500 or 3500 most variable Illumina 450K probes or probes located in CGI that displayed cancer-specific methylation were selected. CIMP-positive tumours identified by the last approach are represented with pink alluvia. Note changes in the flow of CIMP-positive samples from left to right. (**B**) For clustering, probes located in CGI that displayed cancer-specific methylation were selected. Samples were clustered by selecting hierarchical clustering (hc) or k-means (km) or partitioning around medoids (pam). CIMP-positive tumours identified by the last approach are represented with pink alluvia. Note changes in the flow of CIMP-positive samples from left to right.

**Figure 4:**
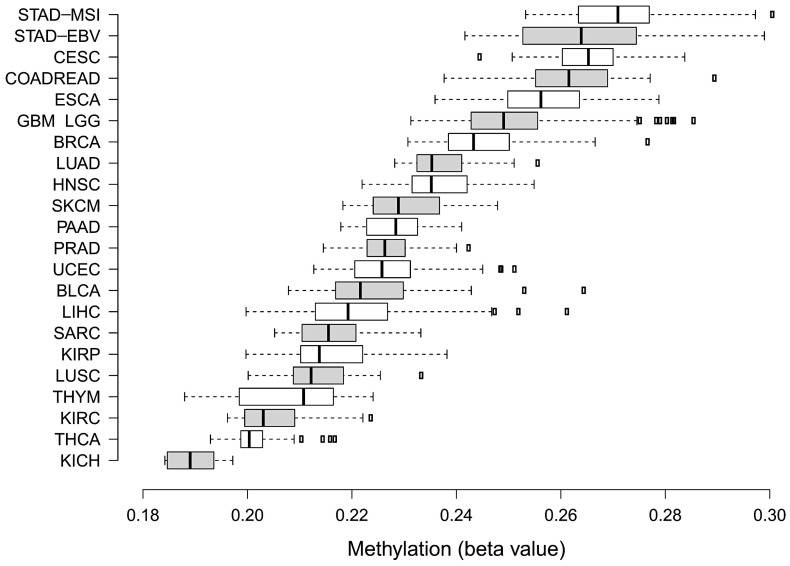
Box plots illustrating differences in average methylation calculated for Illumina 450k array probes located in CGI for CIMP-positive tumours between 23 cancer types. Box plots were ordered in terms of increasing mean CGI methylation from bottom to top. The extraction of CGI methylation together with CIMP assignments were described previously in our work [80].

TCGA has provided the first multi-cancer view of CIMP. However, bioinformatics approaches undertaken by TCGA teams to discover methylation clusters were highly heterogeneous (see Table 1). It must be emphasized that there are still no bioinformatics standards to define CIMP-positive tumours based on methylation array data. Figure 3 illustrates that various feature selection strategies or clustering algorithms may have a significant impact on assigning samples to CIMP-positive clusters, even within a specific tumour type [108]. As such, several groups, including ours, published numerous methylation-centred pan-cancer re-analyses of TCGA data. This ensured the use of comparable approaches to describe methylation clusters in various tumour types. In 2015, Gevaert et al. published one of the first pan-cancer DNA methylation re-analyses using data gathered from 12 tumour types [109]. Apart from confirming the existence of previously reported CIMPs in COAD, LAML and GBM, the authors provided a pan-cancer set of hypo- and hypermethylated genes. In 2017, the same research group used a similar statistical approach to provide a detailed re-discovery and characterisation of the CIMP subtype in HNSCC [110]. In 2015, two independent re-analyses focused on finding commonalities between CIMPs in various cancers were published. A smaller study by Moarii et al. included five tumour types (BLCA, BRCA, COAD, LUAD and STAD) and provided some evidence for common CGI methylation signature for CIMP (consisting of 51 genes); however, it failed to find commonalities concerning gene expression and somatic mutation patterns [111]. A significantly larger study by Sánchez-Vega et al. covered 12 tumour types [112]. They found a common methylation pan-cancer CIMP signature consisting of 89 Illumina microarray probes. Apart from that, Sánchez-Vega et al. searched for CIMP-specific driver mutations, transcriptional programmes and copy-number variations; however, they failed to provide a unifying mechanism for CIMP across these cancer types. In 2018, our group re-investigated 16 TCGA datasets encompassing three data layers (DNA methylation, gene expression and copy-number variations) in 4,688 tumour samples [113]. We found that CIMP tumours in various cancer types are heterogeneous with regard to deregulated pathways and deregulated key transcriptional regulators. Importantly, the average methylation of CGIs in various CIMPs varies considerably (Figure 4). This variability certainly makes comparisons of CIMPs between different tumour types significantly harder.

It is important to note that not all CIMP clusters correspond to clusters obtained during the aggregation of multi-omic data (e.g., gene expression, CNV, miRNA expression, mutational profile) in the same samples and tumour types. In our recent study, we found that only in 9 out of 16 cancer datasets did CIMP clusters significantly overlap with one integrative cluster [113]. This suggests that CIMP often does not represent a molecular phenotype “strong” enough to be able to drive specific molecular clusters. This is a surprising discovery—one that would not have been expected in the past. Given the thousands of CGIs hypermethylated in CIMP tumours, it is hard to believe that such an immense change has little to no influence on tumour phenotype. It is possible that, in the case of “weak” CIMPs, we are just too late with our observation in terms of tumour progression to register the influence of CIMP. For example, a recent genome-wide study has shown that CIMP is present in 27% of intestinal metaplasia which is a pre-malignant condition of the gastric mucosa associated with increased gastric cancer risk [114]. Given that DNA methylation changes are observed very early in carcinogenesis, such “weak” CIMPs could be the driving forces in the initial stages of malignant transformation that are subsequently dominated by other molecular events. A clear example of such a molecular sequence comes from the field of colorectal cancer. For example, a 2006 study of serrated polyps and colon adenomas showed that CIMP develops early, whereas MSI develops late in serrated polyps/adenoma-carcinoma sequence [115]. Consequently, recent 3-year surveillance of small colorectal polyps has demonstrated the presence of CIMP in 32% of samples while none of the polyps displayed MSI [116]. This contrasts with CRC, where about 50% of CIMP samples are also MSI-positive [117]. The CIMP-positive/MSI-positive tumours stand out compared to the CIMP-positive/MSI-negative tumours due to their clinical associations, including poorer prognosis in the latter tumour subgroup [118].

Recent studies explored CIMP in the context of spatial tumour heterogeneity. Verburg et al. applied methylation profiling of multiple sections of diffuse glioma from 16 patients and found only little spatial heterogeneity within each tumour [115]. Similarly, Flatin et al. investigated CIMP in two to four multiregional samples from 30 colorectal primary tumours and found that CIMP status was consistent between multiregional samples. CIMP has also been reported to be concordant between primary CRCs and matched metastases [119].

## 7. Divergent Routes to CIMP

In 2010, Noushmehr et al. identified a subgroup of brain tumours (gliblastoma multiforme; GBM), characterised by outstandingly high levels of CpG island methylation (the so-called G-CIMP) [121]. An important finding of that work was that G-CIMP encompassed almost all cases of tumours with the IDH1 (isocitrate dehydrogenase 1) dominant R132H gain-of-function mutation. Afterwards, Brennan et al. confirmed the association of G-CIMP with IDH1R132H in primary and secondary glioblastoma [122]. In general, G-CIMP patients display a survival advantage over G-CIMP-negative individuals. Two independent studies from 2012 provided evidence that the introduction of IDH1R132H mutant into immortalised cell lines with endogenous IDH1WT induced G-CIMP [123,124]. Later, CIMP-inducing mutations in IDH2 (isocitrate dehydrogenase 2) R172 and R140 hot-spot codons were also discovered [125]. From a mechanistic point of view, mutations of IDH1 and IDH2 result in a high level of oncometabolite 2-hydroxyglutarate (2HG) and a low level of α-Ketoglutarate (α-KG). High 2HG inhibits methylcytosine dioxygenase (TET2) whereas α-KG is an essential co-factor for Jumonji-C histone demethylases [126]. Therefore, histone and DNA demethylases become inhibited, causing an increase in methylation levels. Mutations of IDH1 and IDH2 are also detected in approximately 20% of acute myeloid leukaemia (AML) cases [127]. Similarly to glioblastoma, the presence of IDH1 and 2 mutations correlate with the hypermethylator phenotype (described as A-CIMP) [128]. A-CIMP is associated with longer overall survival in AML. In 2012, Sasaki et al. provided evidence that IDH1R132H is sufficient to induce CIMP in hematopoietic stem cells of knock-in mice harbouring IDH1R132H [129]. IDH1 and 2 mutations have been also found in intrahepatic cholangiocarcinoma (ICC, ~20% cases) [130]. As in the case of the previously described cancer types, IDH mutations in ICC are associated with CIMP and better survival. Significant enrichment with mutated IDH1 and 2 genes has been observed in chondrosarcoma (~50% cases). Consistently, in chondrosarcoma, the presence of IDH1 and 2 mutation associates with widespread deregulation of DNA methylome resulting in CIMP [131]. Unlike in other IDH mutation-dependent CIMPs, a recent report by Nicolle et al. provided no evidence of the prognostic value of CIMP in chondrosarcoma [131]. Mutant IDH1 has also been discovered in a small number of cases of prostate adenocarcinoma (PAC; ~1–2%) [82]. TCGA study suggested that IDH1 mutations define a methylator subtype in PAC; however, the number of CIMP-positive PAC cases is too small to assess the prognostic importance of CIMP in PAC. There is also a cancer type for which the association of IDH mutation with methylator phenotype is not as clear as for tumour types described above. As reported in their TCGA re-analysis, Lauss et al. noted that IDH mutation is present in ~4% of melanoma metastases; however, the enrichment of IDH mutation in the CIMP cluster did not reach statistical significance [132]. An interesting paper that assessed similarities and differences between transcriptome and methylome of IDHMUT and IDHWT in four tumour types (GBM, AML, melanoma and cholangiocarcinoma) has recently been published as well. The authors demonstrated that the overlap of deferentially methylated CpGs between 4 cancer types with IDHMUT is relatively small (n CpGs = 217) and the overlap of deferentially expressed genes is even smaller (n = 1) [133]. These results strongly suggest that IDHMUT imposes heterogeneous effects on the methylome and transcriptome in various cancer types.

Apart from IDH-mutation-induced CIMPs, there is another well-established factor that induces abnormal hypermethylation—the Epstein–Barr Virus (EBV). EBV infection is typically asymptomatic; however, in vitro EBV has a significant B lymphocyte transformation potential and becomes a causal agent of B cell malignancies, including Burkitt’s lymphoma, natural killer cell lymphoma, Hodgkin disease and X-linked lymphoproliferative disease. EBV has also been found to be able to infect epithelial cells and thus has been identified as a causal agent in nasopharyngeal carcinoma and gastric cancers [134]. The CIMP-inducing potential of EBV has been well documented in gastric cancer, in which EBV directly induces the hypermethylation of the host genome following the infection of gastric epithelial cells [135]. More specifically, according to spatiotemporal modelling of EBV infection of normal gastric epithelial cell lines by Matsusaka et al. and Funata et al., EBV induces orchestrated non-random hypermethylation in the proximity of transcription start sites (TSSs) 8 days after infection. The process of hypermethylation is unleashed by two EBV latent proteins—LMP1 and LMP2A—which activate DNA methyltransferase 1 (DNMT1) and transcriptionally silence ten-eleven translocation (TET) demethylases (TET1 and TET2) [136,137]. Collectively, EBV infection in gastric epithelium leads to accelerated transcriptional silencing of many important genes, including tumour suppressors, cell cycle regulators and anti-oncogenic factors. Interestingly, EBV-positive gastric cancers are associated with significant levels of immune infiltration, and consequently, much longer median survival than other gastric cancer molecular subtypes [138].

**Figure 5:**
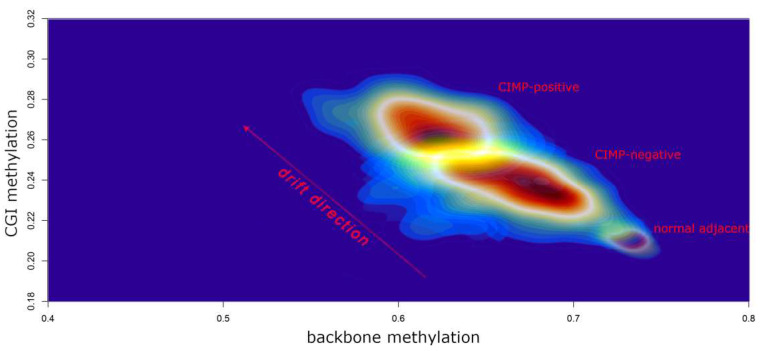
A density plot illustrating increased epigenetic drift (elevated average CGI hypermethylation and decreased average backbone methylation) in CIMP-positive tumours when compared to CIMP-negative tumours and normal adjacent tissue. To design this plot, we downloaded and normalised TCGA methylation data obtained from oesophageal adenocarcinomas, gastric carcinomas and colorectal cancers (819 samples in total). The extraction of CGI methylation and backbone methylation, together with CIMP assignments, were described previously in our work [80]. The colour scale reflects regions with a high sample density (red) and a low sample density (blue). The plot was generated using the MASS R package.

In 2017, our group published a large TCGA data re-analysis of 23 cancer types encompassing over 7200 unique samples [80]. Our primary aim was to assess the differences between tumours characterised by high methylation across CpG islands (CIMP-positive) in comparison to CIMP-negative tumours. We focused on global genomic features, including non-synonymous mutation frequencies and somatic copy number alterations (SCNAs), intergenic regions (backbone) and repetitive sequences (Alu and LINE-1) methylation level as well as epigenetic mitotic-like clock scores and RNAseq-based proliferation markers. We found that more than 90% of CIMP-positive clusters were significantly associated with accelerated epigenetic mitotic clock, demethylation of enhancer sites, backbone and repetitive sequences. In addition, elevated mRNA levels of at least one out of 4 proliferation markers have been revealed in ~70% of CIMP-positive subgroups. These features are specific to a phenomenon commonly defined as “epigenetic drift” that has been previously described in the context of ageing and senescence [139,140]. Therefore, we conclude that the significant departure from the normal DNA methylation pattern observed in CIMP-positive tumours may be fuelled by their high proliferative potential that leads to the expansion of DNA methylation errors [80]. This may explain the general pan-cancer mechanism of establishing CIMP in most tissue types. Figure 5 illustrates the epigenetic drift that we visualised in gastrointestinal cancers. Recently, Tao et al. used a long period culture of mouse intestinal organoids to provide compelling evidence that the accumulation of increased methylation at CGI is due to tissue ageing and an increased number of stem cell divisions [141]. It must be stressed that, despite common mechanistic background, CIMP-positive genomes are likely shaped by various tissue-specific and environmental factors; therefore, we can observe that CIMP-positive tumours display various genomic alternations and/or belong to various expression subtypes even within the same tissue [113].

The use of high-resolution genome-wide methods on DNA methylation in the context of cancer brought important results: in each normal tissue there are CGIs seeded with low-level DNA methylation, which become more methylation-prone in the process of cancerogenesis [142]. This phenomenon seems to be tissue-specific. In the process of DNA methylation expansion in CIMP-positive tumours, these seeded CGIs impose a tissue-specific pattern of DNA methylation. This may explain why few commonalities are found between various CIMPs, even in terms of the DNA methylation patterns.

## 8. CIMP and Targeted Therapy

The fact that frequent hypermethylation of multiple genes had been discovered in many human cancer types has encouraged the idea of therapeutic options that might be based on epigenetically acting drugs [143]. Consequently, shortly after the discovery of CIMP, it has been speculated that these types of tumours may be specifically susceptible to demethylation agents through simultaneous demethylation of multiple genes [23,144]. In addition, the early success of DNA methylation inhibitors and histone deacetylase inhibitors in treating hematologic malignancies (such as myelodysplastic syndrome and acute myeloid leukaemia) have raised hopes for the introduction of these effective treatments in solid tumours [143]. However, few clinical trials in solid tumours have addressed the CIMP status as a target condition, with such trials either being terminated or their outcomes proving inconclusive (Trial ID: NCT03576963, NCT01730586, NCT02786602, NCT01882660) [145]. Several reasons may explain the lack of studies on anti-CIMP targeted therapies. First, CIMP is not routinely tested even in the best-characterised setting—CRC. Second, epigenetic agents are characterised by pharmacokinetic instability and increased toxicities that limit tolerability [146]. Finally, epigenetic trials have often been applied to patients with late stages of disease who have failed multiple prior therapies. Consequently, the overall survival of these patients is expected to be very short even though it is currently accepted that epigenetic therapies require a prolonged application of lower doses. Due to these limitations, there are nine approved epigenetic agents available as of 2021, while most advances in the epigenetic treatment of solid tumours remain a work in progress [146].

Currently, alternative, non-epigenetic treatments are considered for CIMP-positive cases due to the variables associated with CIMP rather than CIMP itself. Direct targeting of the mutant enzyme has been a frequent strategy in patients with IDH-mutant-dependent CIMP [147]. Interestingly, Johannessen et al. has demonstrated in vitro that the use of IDH-mutant inhibitor (AGI-5198) does not eradicate the aberrant histone methylation pattern from the cell line; as such, it is likely that the aberrant DNA methylation pattern associated with CIMP also remains unaltered [148]. Second-generation IDH-mutant oral inhibitors—ivosidenib (AG-120) and enasidenib (AG-221)—are currently approved by the FDA as a therapeutic option for AML [149]. Unfortunately, both drugs displayed low brain penetration in the glioma mouse model. Nonetheless, a new drug called vorasidenib (AG-881) displays improved brain penetration; the results of ongoing trials are promising [150]. As revealed by the ClarIDHy phase 3 study, ivosidenib (AG-120) significantly improved progression-free survival in advanced, IDH1-mutant cholangiocarcinoma and it has been approved by the FDA for advanced or metastatic cholangiocarcinoma [151]. IDH inhibitors are also currently under investigation in phase I clinical trials in chondrosarcoma; however, these early results of ongoing trials are modest in terms of patients’ benefits, with a longer-term analysis required to assess the definitive effect on survival.

Unlike in the case of IDH-dependent CIMPs, indirect therapies in the context of other CIMPs are much less developed. For example, in the colon, gastric (both MSI and EBV) and head and neck cancers, CIMPs are associated with a high neoantigen burden, high degree of lymphocyte infiltration and higher expression of immune checkpoint pathway, which strongly suggests that targeting the immune system by immune-checkpoint inhibitors may lead to improved outcomes in this type of tumours [113,152]. Consequently, in 2020, the FDA approved Keytruda (pembrolizumab) for therapy of unresectable or metastatic MSI-high CRC. Compared to placebo, Keytruda-based therapy extended twice the progression-free survival of patients [153].

## 9. Conclusions and Perspectives

The scientific community’s interest in CIMP has been slowly but surely decreasing ever since its discovery. The epigenetic landscape of CIMP-positive tumours raised hopes for the generalisability of CIMP across tumour types, development of efficient DNA methylation-based diagnostics and treatments of CIMP-positive solid tumours. However, CIMP turned out to be a tissue-specific phenomenon, one that varies across tumour types and is hard to ultimately define, even with the use of modern techniques. Consequently, CIMP failed to become a predictor of clinical outcomes as there is a lack of reproducibility between studies. It might be because our current knowledge of DNA methylation and CIMP remains a patchwork of shattered information obtained from array probes or other low-density techniques. Currently observed development of new methods for long-read whole-genome methylation analysis (e.g., PacBio single-molecule real-time sequencing or nanopore sequencing) may deepen our understanding of CIMP in the near future. Limitations in terms of epigenetic drug delivery and systemic toxicity have also led to unsatisfactory therapeutic efficacy, and it seems that the idea of targeting CIMP-positive tumours by demethylating drugs has been forgotten. In conclusion, it might seem unreasonable to foresee future perspectives for CIMP. Nonetheless, should epigenetic drug delivery and protection against side effects be improved, CIMP tumours may be one of the first targets considered for epigenetic therapies. Assuming that the CIMP pattern is stable across tumour sections, optimal anti-tumour activity is expected for CIMP-positive tumours.

## Figures and Tables

**Table 1:** Data on methodological approaches undertaken to provide methylation clusters in various TCGA publications. Table summarizes 27 TCGA studies on 29 cancer types.

Cancer	Abbreviation	Illumina Platform	CIMP or Highly Methylated Cluster	Probes Selection	No. of Probes Selected	Tumor Purity Addresed	Clustering	Clustering Method	Ref.	Publication Year
Breast adenocarcinoma	BRCA	27K and 450K	Yes	most variable (highest standard deviations)	574	no	NA	RPMM clustering	[81]	2012
Prostate adenocarcinoma	PRAD	450K	Yes	cancer specific hypermethylation (beta value > 0.3)	5000	yes	binary distance clustering/Ward’s method for linkage	hierarchical clustering	[82]	2015
Bladder urothelial carcinoma	BLCA	450K	Yes	cancer specific/promoter and CpG island associated (beta value > 0.3)	31,249	yes	binary distance clustering/Ward’s method for linkage	hierarchical clustering	[83]	2014
Ovarian serous cystadenocarcinoma	OV	27K	Not reported	most variable (highest standard deviations)	858	no	K-means clustering/Euclidean distance	consensus clustering	[84]	2011
Colorectal adenocarcinoma	COAD and READ	27K	Yes	most variable (highest standard deviations)	2758	no	NA	RPMM clustering	[85]	2012
Lung adenocarcinoma	LUAD	27K and 450K	Yes	promoter and CpG island associated/1.0% of most variable	not provided	no	PAM clustering/Euclidean distance	consensus clustering	[86]	2014
Lung squamous cell carcinoma	LUSC	27K and 450K	Not reported	most variable (highest standard deviations)	8228	no	PAM clustering/Euclidean distance	consensus clustering	[87]	2012
Uterine corpus endometrial carcinoma	UCEC	27K and 450K	Yes	most variable (highest standard deviations)	785	no	NA	RPMM clustering	[88]	2013
Acute Myeloid Leukemia	AML	450K	Yes	most variable (highest standard deviations)	1000	no	Euclidean distance clustering/Ward’s method for linkage	hierarchical clustering	[89]	2013
Glioblastoma	GBM	GoldenGate and 27K	Yes	most variable (highest standard deviations)	370	no	K-means clustering/Euclidean distance	consensus clustering	[90]	2008
Stomach adenocarcinoma	STAD	27K and 450K	Yes	cancer specific hypermethylation	1375	yes	binary distance clustering/Ward’s method for linkage	consensus clustering	[51]	2014
Thyroid carcinoma	THCA	450K	Yes	most variable 1.0% of probes	not provided	no	PAM clustering/Euclidean distance	consensus clustering	[91]	2014
Head and neck squamous cell carcinoma	HNSC	450K	Yes	most variable 1.0% of probes	not provided	no	PAM clustering/Euclidean distance	consensus clustering	[92]	2015
Skin cutaneous melanoma	SKCM	450K	Yes	most variable 1.0% of probes	not provided	no	PAM clustering/Euclidean distance	consensus clustering	[93]	2015
Brain lower grade glioma	LGG	450K	Yes	cancer specific hypermethylation (beta value > 0.3)	11,977	yes	binary distanceclustering/Ward’s method for linkage	hierarchical clustering	[94]	2015
Kidney renal papillary cell carcinoma	KIRP	27K and 450K	Yes	cancer specific hypermethylation/most variable	not provided	no	Ward’s method	hierarchical clustering	[95]	2016
Adrenocortical carcinoma	ACC	450K	Yes	promoter and CpG island associated/1.0% of most variable	not provided	no	PAM clustering/Euclidean distance	consensus clustering	[96]	2016
Uterine carcinosarcoma	UCS	450K	Yes	cancer specific hypermethylation/most variable	5000	no	Ward’s method for linkage	hierarchical clustering	[97]	2017
Cholangiocarcinoma	CHOL	450K	Yes	cancer specific hypermethylation (beta value > 0.3)	37,743	yes	binary distance clustering/Ward’s method for linkage	hierarchical clustering	[98]	2017
Cervical cancer	CESC	450K	Yes	promoter and CpG island associated/1.0% of most variable	not provided	no	PAM clustering/Euclidean distance	consensus clustering	[99]	2017
Hepatocellular carcinoma	HCC	450K	Yes	cancer specific hypermethylation (beta value > 0.3)	37,848	yes	binary distance clustering/Ward’s method for linkage	hierarchical clustering	[100]	2017
Uveal melanoma	UVM	450K	Yes	most variable 1.0% of probes	3859	no	PAM clustering/Euclidean distance	consensus clustering	[101]	2017
Pancreatic adenocarcinoma	PAAD	450K	Yes	cancer specific hypermethylation (beta value > 0.25)	31,956	yes	binary distance clustering/Ward’s method for linkage	consensus clustering	[102]	2017
Sarcoma	SARC	450K	Yes	most variable 1.0% of probes	not provided	no	PAM clustering/Euclidean distance	consensus clustering	[103]	2017
Thymoma	THYM	450K	Not reported	most variable 1.0% of probes	not provided	no	PAM clustering/Euclidean distance	consensus clustering	[104]	2018
Testicular cancer	TGTC	450K	Yes	most variable (standard deviation >= 0.26)	9614	yes	Euclidean distance clustering/Ward’s method for linkage	hierarchical clustering	[105]	2018
Malignant pleural mesothelioma	MPM	450K	Not reported	most variable 1.0% of probes	not provided	no	PAM clustering/Euclidean distance	consensus clustering	[106]	2018
Oesophageal carcinoma	ESCA	450K	Yes	cancer specific hypermethylation (beta value > 0.25)	not provided	yes	binary distance clustering/Ward’s method for linkage	consensus clustering	[107]	2017

## Data Availability

Data supporting reported results are publicly available at Genomic Data Commons (https://portal.gdc.cancer.gov/, accessed date: 10 October 2020).
